# Experimental evaluation of an intraoperative‐imaging based workflow for electron beam radiotherapy of pancreatic cancer using in situ dosimetry

**DOI:** 10.1002/acm2.70556

**Published:** 2026-03-27

**Authors:** Charoula Iliaskou, Mark Gainey, Michael Kollefrath, Siegmar Kuhn, Vasilios Boronikolas, Andreas R. Thomsen, Dietrich A. Ruess, Anca‐Ligia Grosu, Dimos Baltas

**Affiliations:** ^1^ Division of Medical Physics Department of Radiation Oncology Faculty of Medicine Medical Center – University of Freiburg University of Freiburg German Cancer Consortium (DKTK), partner site DKTK‐Freiburg Freiburg Germany; ^2^ Department of Radiation Oncology Medical Center‐University of Freiburg Faculty of Medicine University of Freiburg German Cancer Consortium (DKTK), partner site DKTK‐Freiburg Freiburg Germany; ^3^ Department of General and Visceral Surgery Medical Center Faculty of Medicine University of Freiburg, German Cancer Consortium (DKTK), partner site DKTK‐Freiburg Freiburg Germany

**Keywords:** CBCT, electron beams, image‐guided, in situ dosimetry, in vivo dosimetry, IOERT, Monte Carlo algorithm, pancreas, TLD‐100 dosimetry

## Abstract

**Purpose:**

The aim of this study is to perform an experimental evaluation of an imaging‐based intraoperative electron beam radiotherapy (IOERT) and in vivo dose verification workflow for pancreatic cancer on a porcine cadaver.

**Materials and methods:**

The Imaging Ring m (ImR) mobile cone‐beam computed tomography (CBCT) scanner (medPhoton GmbH, Salzburg), the Radiance (GMV, Tres Cantos, Madrid, Spain) treatment planning system (TPS) and the Mobetron (IntraOp Medical Inc, Sunnyvale, CA, USA) mobile linear accelerator (LINAC) were used. Cylindrical thermoluminescent dosimeters (TLD‐100) were employed for in situ dose measurements. ImR calibration data were acquired and imported into Radiance for CT table commissioning. The porcine cadaver was immobilized using standard radiotherapy (RT) equipment and scanned preoperatively with a SOMATOM Go.Open Pro CT scanner (Siemens Healthineers AG, Forchheim, Germany) to obtain a reference abdominal CT image. Subsequently, a surgical procedure was performed to expose the pancreas, and a dedicated TLD‐based dosimetry system was secured on its surface. The 5 cm diameter/30°‐bevel IOERT plastic applicator was positioned over the dosimeters and intraoperative CBCT images were acquired. Treatment was delivered using a 9 MeV electron beam, prescribing 10 Gy to the distal 90% isodose depth. The intraoperative CBCT images were imported into Radiance, where the applicator was positioned based on imaging, relevant anatomy was contoured and three‐dimensional (3D) dose distributions were calculated using a Monte Carlo (MC) algorithm and compared to the TLD measurements.

**Results:**

ImR CBCT calibration scans yielded CBCT numbers consistent with reference data. Image quality was sufficient for clear visualization of the applicator and TLD‐based dosimetry systems without significant artifacts; however, soft‐tissue contrast was limited for clear determination of pancreatic tissue and important neighboring vessels. Due to observed tissue extension within the applicator, dose calculations were adjusted to begin inside the applicator volume. In situ TLD dose measurements agreed with MC‐calculated doses within 3%.

**Conclusions:**

The developed image‐guided pancreatic IOERT workflow was successfully simulated under near‐clinical conditions using a porcine cadaver model. Agreement between in situ TLD measurements and MC dose calculations was within the accepted tolerance of ±5%, supporting further clinical implementation.

## 1 INTRODUCTION

Intraoperative electron beam radiotherapy (IOERT) delivers a single high‐dose irradiation directly to residual tumor cells immediately after surgical excision. Currently, mobile linear accelerators (LINACs) operate at nominal electron energies ranging from 4 to 12 MeV. The electron applicators used to collimate the beam are available in cylindrical or rectangular shapes, with various sizes and bevel angles.^1^ In standard IOERT clinical protocols, the dose is prescribed to the distal depth of the 90% isodose line of the depth‐dose curve in water. The electron energy is selected such that this isodose fully encompasses the tumor bed in depth (z‐direction). The extend of the tumor bed is visually assessed after tumor removal. The applicator size and bevel angle are then chosen to ensure adequate coverage in the transverse plane (x,y directions), typically including an additional 2 cm margin.^2^, ^3^, ^4^, ^5^, ^6^ The required monitor units (MU) are calculated using tabulated data generated during LINAC commissioning. Subsequently, the surgical team carefully positions the applicator to ensure that surrounding healthy tissues remain outside the treatment field. Moreover, in vivo dosimetry is recommended as a quality assurance (QA) tool and has revealed significant deviations from the expected doses in several centers.^6^, ^7^, ^8^, ^9^ These discrepancies have most often been attributed to misalignments between the applicator and either the dosimeters or the target. As a result, uncertainties in dose delivery to the tumor bed and adjacent organs may arise. The lack of a robust dose–volume information in current IOERT protocols,^6^ may ultimately compromise treatment quality. Consequently, considerable efforts in recent years have focused on the introduction of three‐dimensional (3D) imaging for applicator positioning and treatment planning. These advances aim to improve the accuracy of dose delivery and to overcome existing limitations.^6^, ^7^ In parallel, the development of fast Monte Carlo (MC) dose calculation techniques has enabled treatment planning to be performed during clinical procedures without compromising reliability or accuracy.^8^ An example is Radiance, currently the only commercially available TPS for IOERT, which integrates the Dose Planning Method (DPM) fast MC dose calculation algorithm.^9^ At the time of its original development, imaging technologies were less advanced than those available today. As a result, the system was designed primarily for preoperative virtual planning based on computed tomography (CT) volumetric images. In this workflow, a virtual surgical frame simulated the operative region, enabling virtual applicator placement on the preoperative CT images. However, pre‐ and postoperative anatomy differ substantially. Intraoperative imaging has therefore been shown to be essential, as preoperative planning alone leads to unreliable dose–volume results.^10^ Therefore, recent research has focused on integrating intraoperative imaging to enable reliable treatment control and planning. Investigated approaches include surface scanning techniques,^11^ ultrasound (US) imaging,^12^, ^13^ transferring the patient from the operation room (OR) to the CT simulator,^10^ and the use of various CT and cone‐beam computed tomography (CBCT)^14^ as well as fluoroscopic CBCT devices.^15^, ^16^ Nevertheless, none of these approaches has yet established a robust foundation for imaging in IOERT, as they do not fully meet the requirements for routine clinical integration. It is crucial to implement a time‐efficient imaging procedure that minimizes disruption to the surgical workflow under anesthesia constraints. In parallel, sufficient and reproducible image quality along with reliable CBCT numbers must be ensured to allow accurate dose calculations. Recently, a mobile CBCT scanner, the Imaging Ring m (ImR; medPhoton, Salzburg, Austria), has become commercially available, enabling image‐guided brachytherapy and IOERT. The ImR differs from previously tested fluoroscopic or CBCT devices for intraoperative imaging (such as O‐arms) by featuring a wide bore diameter of 121 cm, a flat‐panel detector (FPD) with an active area of 43.2 × 43.2 cm^2^ (17 × 17 in^2^), nine degrees of freedom of movement, and easy monitoring via a mobile computer‐console. These features enhance practicality and facilitate patient positioning within the OR. To date, the first groups worldwide to employ the ImR have published extensive technical evaluations of it. They focused primarily on its use in adaptive Brachytherapy.^17^, ^18^, ^19^, ^20^, ^21^, ^22^ In addition, its initial clinical application for treatment planning in IOERT for rectal cancer using the Radiance TPS has been reported,^23^ demonstrating the feasibility of this workflow for clinical implementation. However, unlike image‐guided or adaptive EBRT, for which CBCT ‐based dose calculations have been thoroughly validated,^24^, ^25^, ^26^, ^27^, ^28^, ^29^ this is not yet the case for IOERT. The image quality, reliability and consistency of CBCT numbers in IOERT set‐ups still require quantitative evaluations and standardization. In our department, we aim to implement an image‐based planning and dose delivery IOERT protocol for patients with pancreatic adenocarcinoma, extending the image‐guided IOERT applications to this disease site. To this end, an intraoperative treatment planning workflow based on in‐room CBCT and MC‐based dose calculations, followed by in vivo dosimetry has been developed. A pilot phase clinical trial including twenty patients has been registered. The primary endpoints are documentation of the delivered prescribed doses and individualized dose prescription. Building on previous studies from our group addressing TPS accuracy and TLD reliability at IOERT dose levels, as well as published data on the ImR CBCT scanner, the present work simulates the developed workflow on a porcine cadaver. The experiment was conducted under real IOERT conditions and serves as an end‐to‐end evaluation prior to clinical translation. In this context, results obtained from in vivo dosimetry are referred to as in situ dosimetry, reflecting their use in a nonliving model. Finally, practical considerations related to the current equipment and future perspectives are discussed.

## 2 MATERIALS AND METHODS

### 2.1 General description of the developing clinical workflow

IOERT treatments are delivered using a Mobetron (IntraOp Medical Inc, Sunnyvale, CA, USA) mobile LINAC equipped with flat‐end and 30°‐beveled applicators with diameters ranging from 50 to 90 mm. The applicators are made of x‐ray compatible material, polyoxymethylene (POM). The CBCT imaging system is the portable ImR scanner (medPhoton, Salzburg, Austria). Treatment planning is performed using Radiance version 4.08.2, employing the MC heterogeneity algorithm, which accounts for tissue heterogeneities. Surgical procedures are conducted on a dedicated OR table, the TS7500 Carbon FloatLine (OR table TruSystem 7500 with the table top Carbon FloatLine, Baxter Medical Systems GmbH + Co. KG, Saalfeld, Germany), which supports intraoperative CT and Magnetic Resonance Imaging (MRI). The Mobetron is typically parked outside the main surgical room and moved in on the day of the operation, when daily or additional pre‐treatment checks are performed. Similarly, the ImR scanner is brought into the OR the day before surgery. The morning quality control (QC) check is performed prior to the operation. Given the limited capacity of the OR, the parking positions of the IOERT table, irradiation, imaging devices and surgical equipment were optimized to accommodate the available space and the planned workflow. Proper patient positioning on the table and correct placement of the applicator holder arm are critical to allow the ImR to approach the patient without collision risks, while ensuring that the detector field of view (FOV) adequately covers the abdominal region. At the same time, sufficient clearance is maintained for the Mobetron to move underneath the table and align with the applicator using its camera‐based soft‐docking system.

The procedure begins with an anatomical assessment of the resected volume and the target‐area by the surgeon and radiation oncologist to define the prescription dose, electron energy, and applicator type. Subsequently, TLDs‐rods are inserted into the sterile set‐up. The surgeon then positions the sterile detector‐ system over the tumor bed and places the applicator on top. In this configuration, the TLDs measure the entrance dose to the tumor bed (PTV), a clinically relevant region that is typically associated with larger dosimetric uncertainties. The TLD placement was determined in consultation with the surgeons to ensure accurate dosimetry while minimizing intervention in the surgical cavity and maintaining sterility. The surrounding non‐treatment areas are then covered with sterile drapes, after which CBCT image acquisition is initiated. If the images reveal incorrect applicator positioning, adjustments are performed and the scan is repeated. The acquired images are exported to the Radiance TPS, where MC‐based dose calculations are performed according to the dose prescription. The resulting MU values are validated against manually calculated values before being transferred to the treatment mode of Mobetron console for dose delivery. In Radiance version 5, energy mixing is also available; alternatively, two separate plans with different energies can be used to achieve the required target dose. The physician then contours the regions of interest and reviews the calculated dose distributions. In parallel, the Mobetron is moved into position and soft‐docking is initiated to couple the LINAC with the applicator placed on the patient. Once alignment is complete, irradiation is delivered. After treatment, the TLD‐based in vivo system is collected in sterile disposable bags and transported to the laboratory for analysis. Finally, the calculated doses are validated against the in vivo measurements.

### 2.2 Electron density calibration of ImR for treatment planning

The acquisition of calibration curves relating CT‐CBCT numbers to physical density (g/cm^3^) is required to enable MC simulations on CT‐CBCT images using the MC heterogeneity algorithm. The acquired calibration tables for the imaging system were imported into the LINAC Configuration Tool interface of the Radiance TPS. After user approval of the input tables, a stoichiometric calibration, following the method of Schneider et al.^30^ is conducted. This procedure maps Hounsfield Units (HU) values to known materials in the TPS library and establishes a continuous relationship between HU and physical density (g/cm^3^), with 1‐HU increments over the range from −1000 to 2500 HU. The electron density (ED) phantom used for calibration was the CIRS 062MA (CIRS Model 062MA electron density phantom, CIRS, Inc., Norfolk, VA, USA), with dimensions of 40 × 30 × 16.5 cm^3^ (W × H × D). The material composition and stoichiometric data of the phantom were integrated into Radiance. In this phantom, a slab containing cylindrical inserts of various physical densities (g/cm^3^) was positioned between two homogeneous slabs of Plastic Water‐LR (15 keV – 8 MeV) to simulate full scatter conditions (Figure 6a). Two material inserts with identical physical density were placed at different locations within the phantom, one in the inner and one in the outer cylinder. In this study, calibration tables were acquired with the ImR using custom protocols (Pr) at 110 kV_p_ and 120 kV_p_ (Table 1: Pr‐110 and Pr‐120). These protocols were defined after several test acquisitions using different imaging and reconstruction settings. The active detector settings of the FPD were 1440 × 1440 px^2^, 11.9 fps (84 ms) and gain 3 (114e^−^/ADU). The CIRS 062MA body phantom was scanned using both a large field of view (LFOV; 32 × 41 cm^2^, Dual Short scan (2 × 180°+ div)) and a small field of view (SFOV; 23.5 × 24.8 cm^2^, full scan) under identical acquisition settings. In our workflow, SFOV is generally preferred because it minimizes the inclusion of surgical tools in the field of view (FOV) which could introduce significant scatter. Therefore, while LFOV was used to ensure full coverage of the phantom body for calibration curve acquisition, an additional scan was performed in SFOV, covering only the phantom's inner cylinder. The inner cylinder contained the same material inserts as the outer cylinder (Figure 6a). This approach allowed us to investigate whether a dedicated calibration curve is required for the SFOV scans. LFOV Dual Short scans were used instead of full offset scans (also available mode in the ImR device), as they provided better CBCT number uniformity. In addition, the ED phantom was scanned on our clinical planning CT scanners (Philips Big Bore and Somatom Go.Open Pro) using the standard pelvis protocol at 120 kV_p_, to obtain reference data. For data analysis, a volume of interest (VOI) with 2 cm diameter and 2 cm length was contoured in Eclipse™ v15.6 TPS to extract CBCT number values and their standard deviation (*k* = 1) for each material insert. For each insert, a single mean CBCT or CT number value was calculated by averaging the measurements obtained at the two different positions within the ED phantom. The standard deviation (*k* = 1) of this mean value was determined using the error propagation method.

**TABLE 1:** Custom‐defined ImR imaging protocols commonly used in clinical practice.

Protocol	Acquisition time (s)	Tube voltage (kV_p_)	X‐ray preset	Focal spot (mm)	Prefilter	Kernel	Object scatter cut‐off	Mode
Pr‐110	65	110	14 mA, 17 ms	0.6	0.5 mm Cu	HAM (0.8)	+1.00	Modulated continuous
Pr‐120	66	120	20 mA, 20 ms	0.3	0.5 mm Cu	SL (+1.00)	+0.9	Constant Pulsed

### 2.3 Preparation of the porcine cadaver and application of the workflow

**FIGURE 1:**
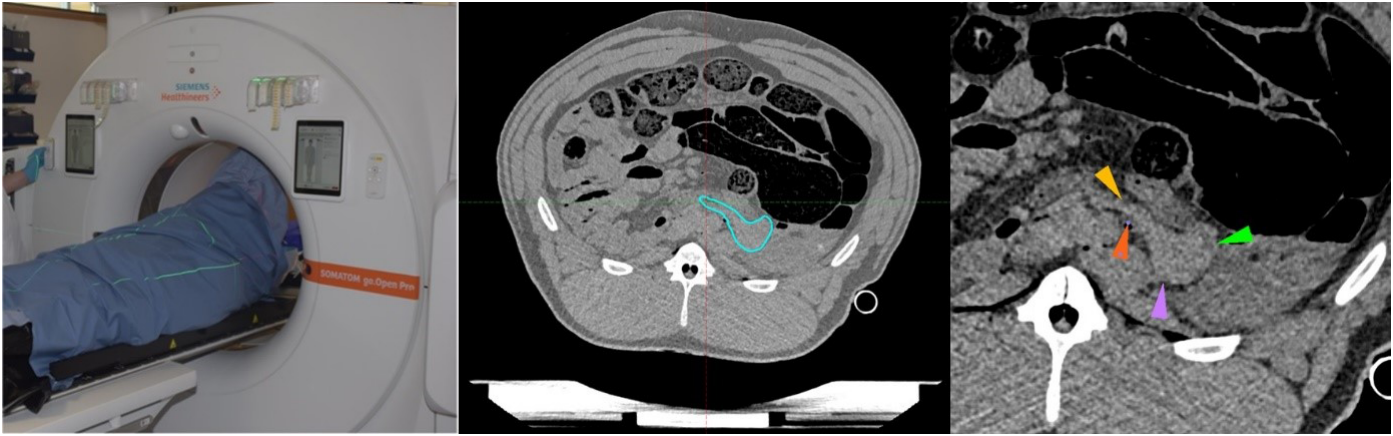
CT scan of the pig cadaver in Somatom Go.Open Pro, visualization of the reconstructed image for the definition of pancreatic tissue in the animal. Colorful arrows point to the pancreatic tissue.

**FIGURE 2:**
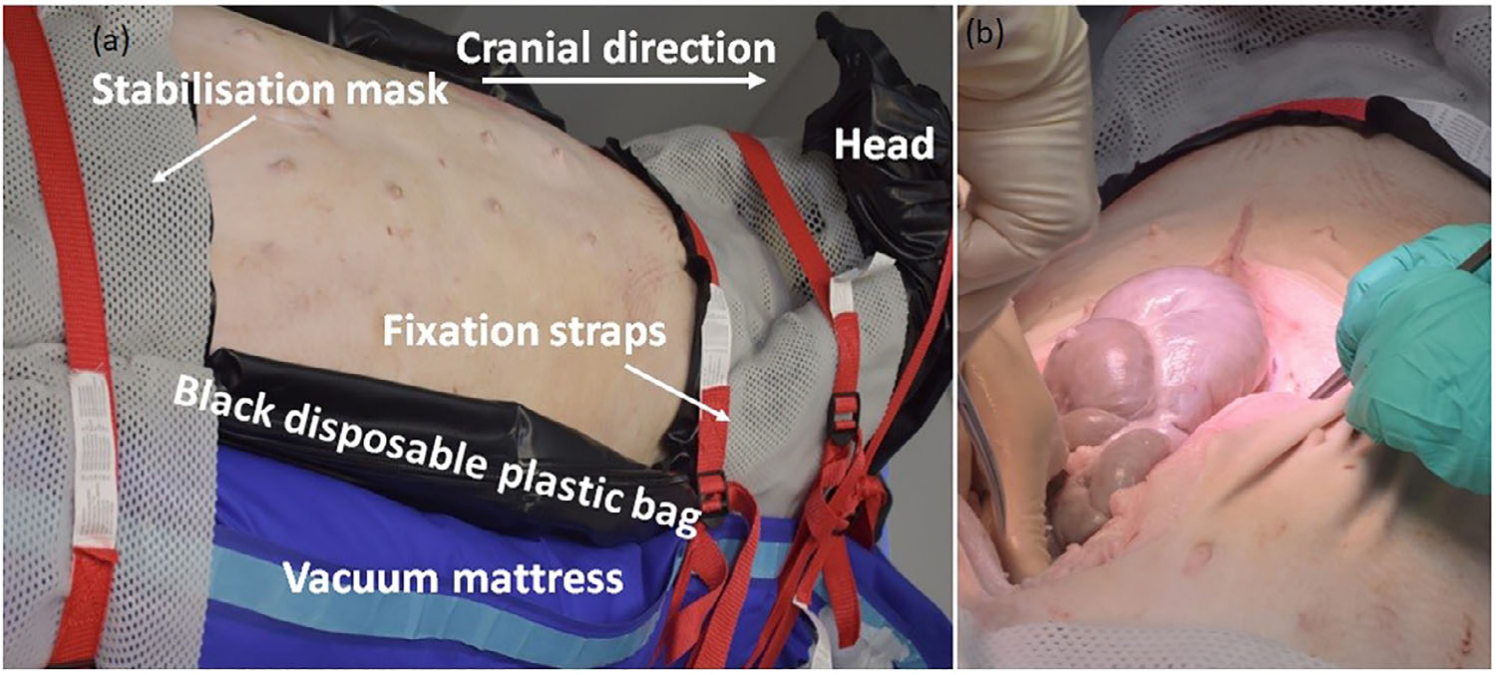
(a) The stabilization of the porcine cadaver on an air mattress for later positioning on the treatment IOERT couch and (b) the beginning of the abdominal surgical procedure.

A porcine cadaver was used to apply the developed intraoperative imaging‐based workflow. The porcine cadaver was transported in a disposable plastic body bag directly from a local abattoir to our brachytherapy suite, which is approved by the Radiation Protection Agency for experimental IOERT‐LINAC procedures. For positioning on the treatment couch, a vacuum mattress commonly for patient immobilization in external beam radiotherapy was placed on the dedicated IOERT table. Custom‐made straps fabricated from thermoplastic mask material were used to support the body and ensure an immobile posture. Additionally, two fixation straps were employed to further secure the cadaver in place (Figure 2a). Prior to operation, the cadaver was scanned on the Somatom Go.Open Pro planning CT to obtain a preoperative image. This step simulated a clinical scenario in which a patient would undergo diagnostic imaging or have received prior EBRT. Pancreatic tissue was identified on the CT image (Figure 1).

**FIGURE 3:**
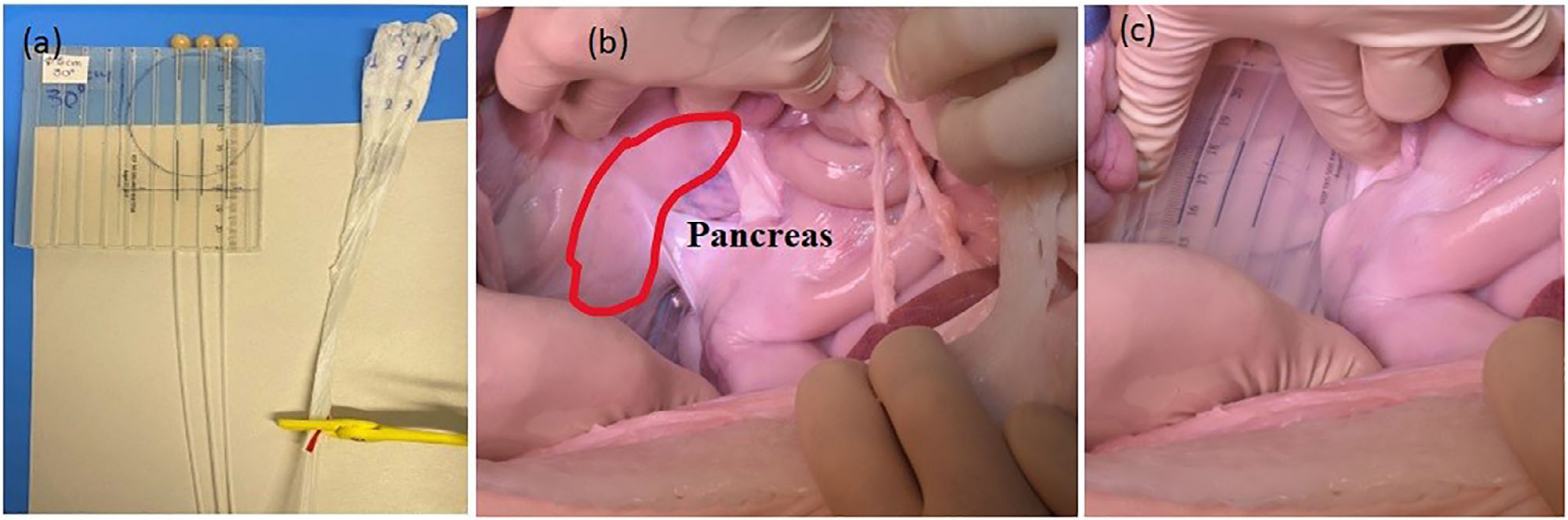
(a) The two prepared TLD set‐ups for the in vivo (in situ in this study) dosimetry (the flap material with three catheters with loaded TLDs on the left and the three catheters loaded with TLDs wrapped in a foil on the right), the flap was cut as indicated by the vertical line touching the drawn circle that represents the diameter of the applicator, (b) open abdominal area of the pig cadaver with the pancreas exposed and (c) placement of the flap‐based set‐up over the pancreas.

**FIGURE 4:**
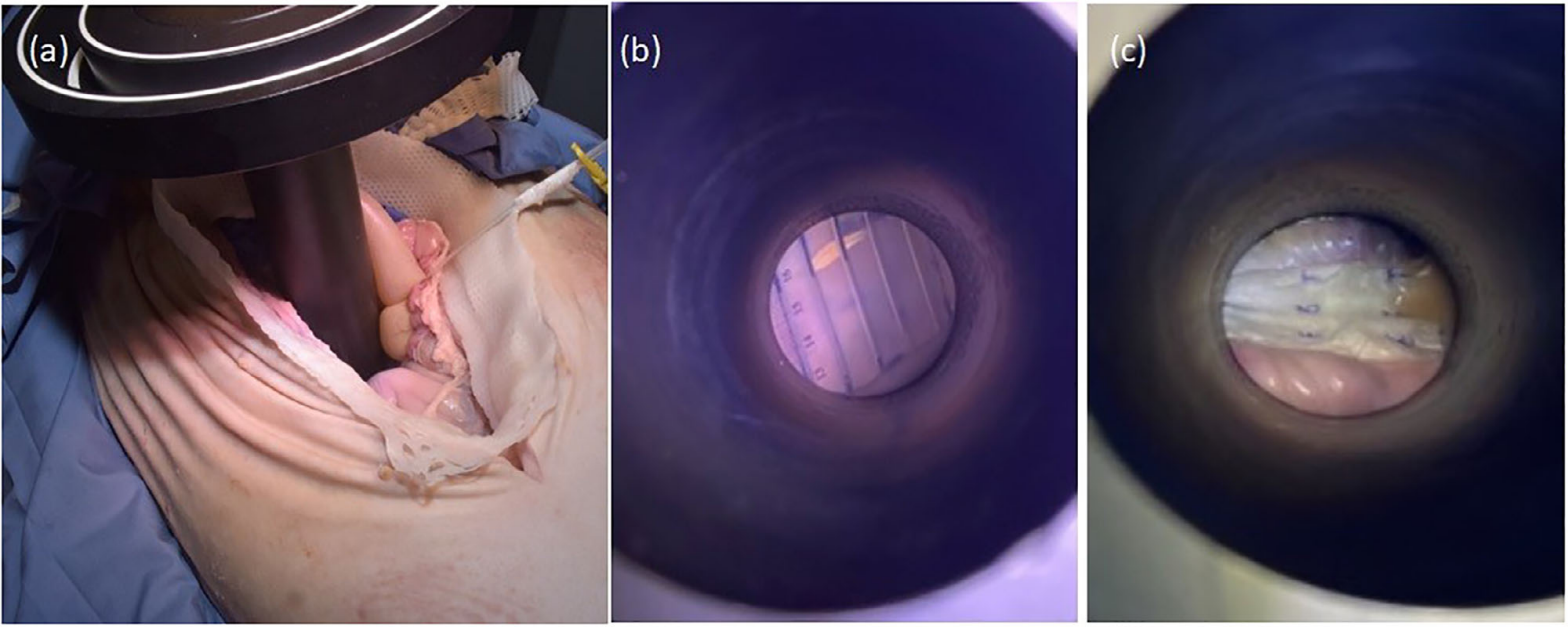
(a) Positioning of the IOERT applicator over the TLD‐based dosimetry system, (b) applicator view of the flap‐based set‐up and (c) applicator view of the catheter set‐up wrapped in foil.

**FIGURE 5:**
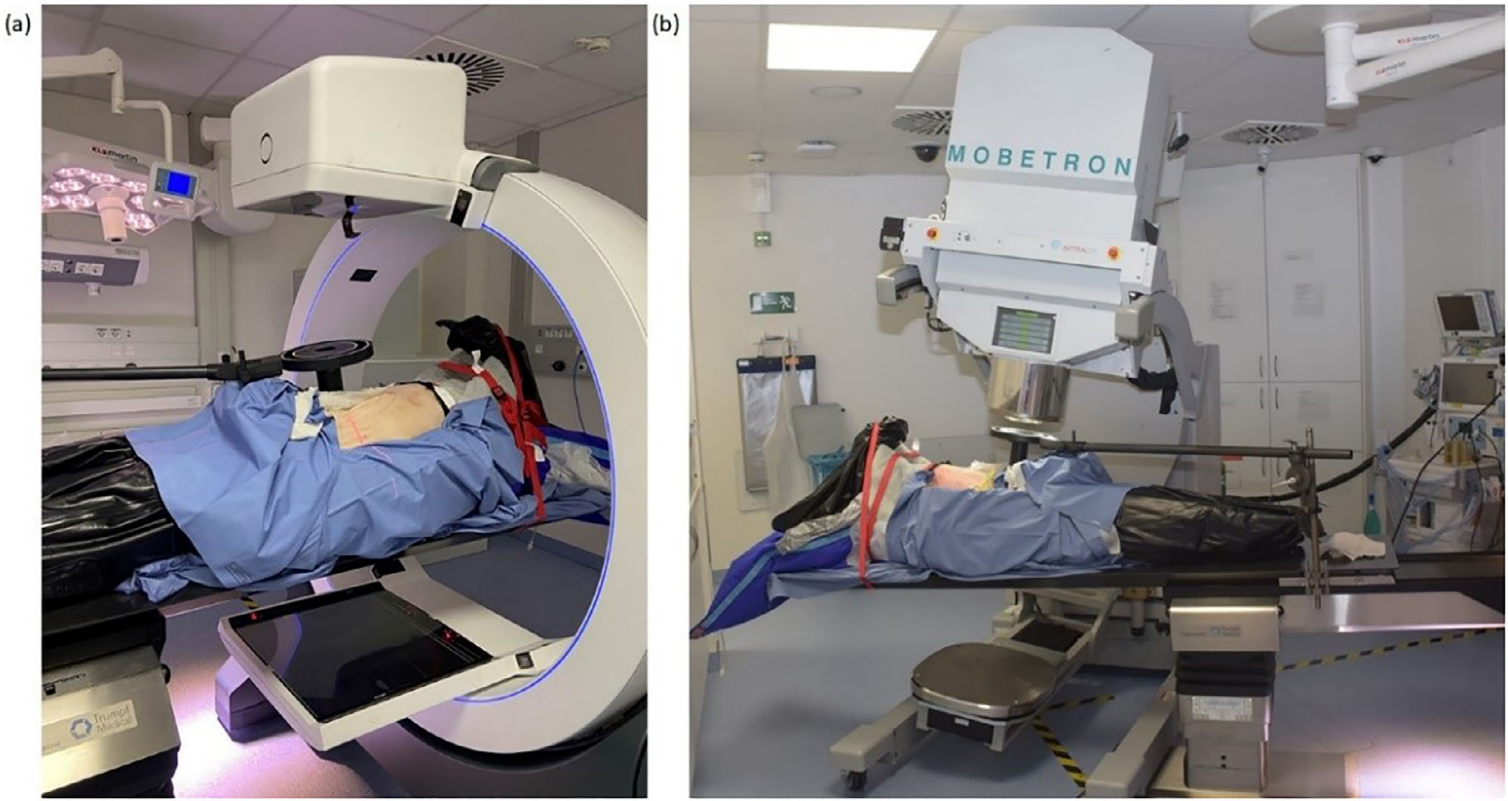
(a) ImR CBCT intraoperative acquisition and (b) treatment delivery with Mobetron LINAC showing the air gap to the clinical applicator.

The porcine cadaver underwent an abdominal surgical procedure (Figure 2b) to expose the pancreatic splenic lobe (pancreatic tail) in situ (Figure 3b) and to allow placement of the prepared TLD‐based dosimetry set‐ups directly on its surface (Figure 3a, c). Following surgical opening of the abdominal cavity, a thermoplastic mask was used to maintain the opening (Figure 4a), replacing the conventional metallic surgical ring. Two dosimetry configurations were evaluated. The first configuration employed a flap material (HAM applicator manufactured by BEBIG Medical, Berlin, Germany) containing three catheters, each loaded with five TLD dosimeters. The second consisted of three catheters, also containing five TLDs each, enclosed within a sterile foil. These set‐ups have been described in detail in our previous publication^31^ and are illustrated in Figure 3a. CBCT scans were acquired for each TLD set‐up; consequently, two separate plans were generated in Radiance. The ImR was brought into position to acquire intraoperative CBCT images. The ImR protocol at 110 kV_p_ was applied to the catheter set‐up wrapped in sterile foil to enhance contrast between the catheters and the surrounding tissue by using lower incident photon energy. The 120 kV_p_ protocol was used for the flap‐based set‐up. To ensure proper alignment and avoid collision warnings before imaging, the treatment couch was marked to indicate the correct positioning of the pig's abdominal region and the placement of the applicator holder. This approach enabled accurate and safe set‐up of the ImR relative to the porcine cadaver. As shown in Figure 5a, b, the IORT couch was fully extended, with the applicator arm‐holder positioned at its maximum extension away from the abdominal region. This configuration enabled a safe set‐up for successful image acquisition and irradiation. After CBCT image acquisition (Figure 5a) the couch was moved to the Mobetron treatment position within the operating room (Figure 5b). The applicator was optically coupled to the Mobetron treatment head using an auto‐docking system guided by integrated cameras. These cameras ensure a fixed air gap between the LINAC head and the applicator. Both irradiations were delivered using a 9 MeV electron beam, with a prescribed dose of 10 Gy at the depth of the distal 90% isodose line, corresponding to the standard prescription used in our department for routine pancreatic IOERT treatments. Based on our tabulated data, this corresponded to 786 MU.

After irradiation, the intraoperative CBCT images (Figure 7) were imported into the Radiance TPS (version 4.08.2). Image quality was assessed by evaluating the visibility of structures of interest, including the pancreas‐ PTV, the TLD‐based dosimetry set‐up, the applicator walls and the surrounding tissue. For Radiance dose calculations, a 9 MeV electron beam was prescribed to deliver 10 Gy to the distal 90% isodose depth, with no further normalization of the dose distribution. Calculations were performed using a 2 × 2 × 2 mm^3^ dose grid and a 1% MC uncertainty tolerance. The rationale for these parameter options has been discussed in our earlier publication on evaluation of the Radiance MC algorithm.^32^ Because tissue was observed inside the IOERT applicator, the option to calculate dose within the applicator was activated. The virtual applicator was positioned such that dose calculations began at the point where tissue first appeared. Further dosimetric analysis, including dose statistics, was carried out in Eclipse, which currently offers a broader set of tools for such evaluations. Regarding the TLD dosimetry, the dosimeters were carefully removed from the catheters to prevent contamination and placed back in the annealing tray for the post‐irradiation and pre‐reading annealing to 100°C for approximately 20 min. The TLDs were then read using a RADOS TLD reader. Dose values were calculated using in‐house Excel 2016 (Microsoft Office) based tools incorporating calibration data. A detailed description of the TLD dosimetry methodology, specifically developed for in vivo dose measurements in IOERT, is provided in our previous publication.^31^

## 3 RESULT

### 3.1 Electron density calibration of ImR for treatment planning

**FIGURE 6:**
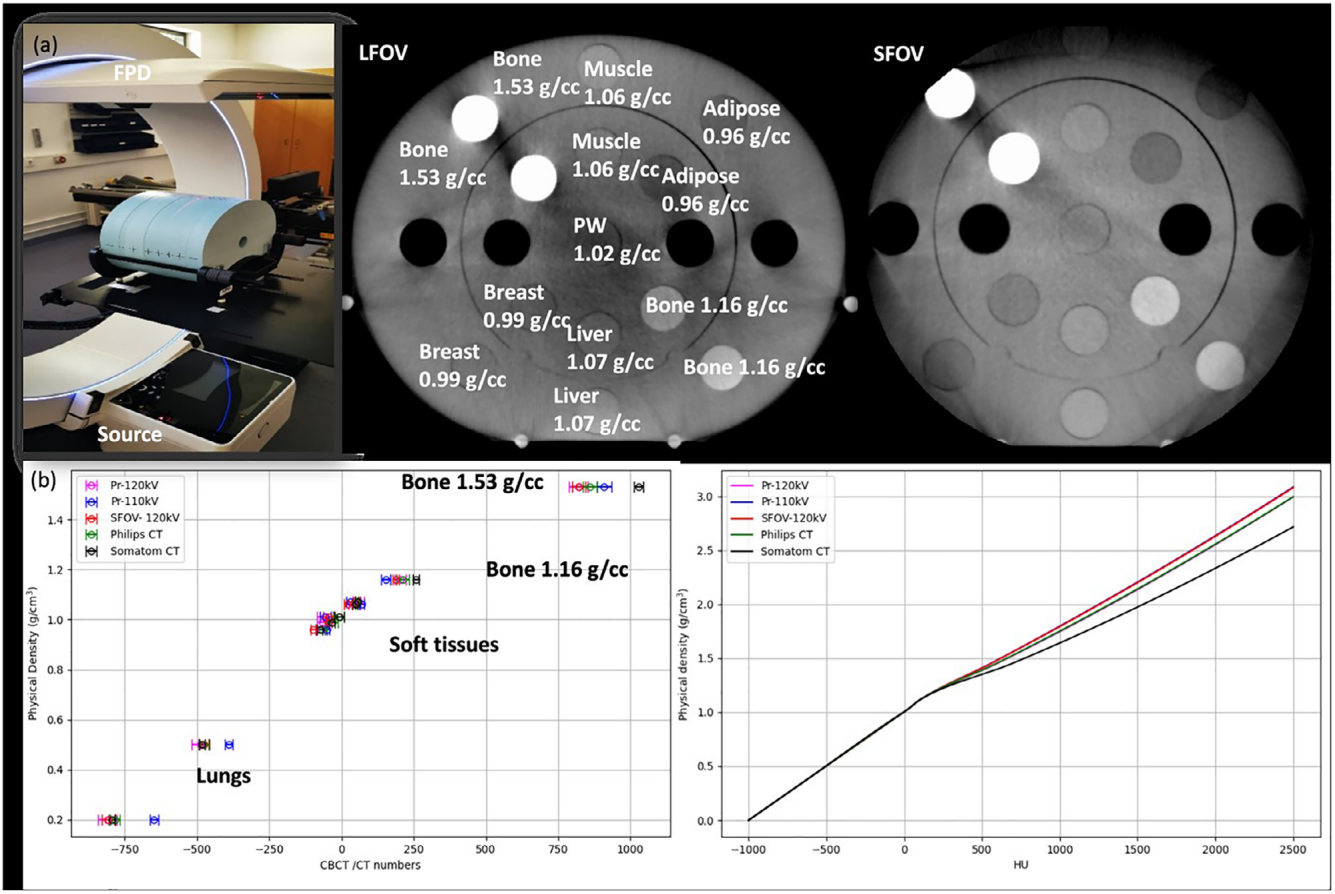
(a) The CIRS 062MA ED phantom scanned with ImR, showing a transverse view of the central slice acquired in LFOV mode, (b) calibration curves for the ImR imaging protocols and the two planning CT systems (Philips Big Bore CT and SOMATOM Go.Open Pro), together with the corresponding LUTs generated in the Radiance TPS.

The set‐up for scanning the CIRS 062MA ED phantom is shown in Figure 6a. Both acquisition modes LFOV and SFOV demonstrated very good performance, yielding CBCT numbers consistent with the expected values from reference CT data (Figure 6b). Due to truncation effects in SFOV scans, only the inner cylinder CBCT‐number values were considered in the analysis. The resulting calibration curves and LUT are shown in Figure 6b. The LUTs generated in Radiance were identical for both LFOV and SFOV protocols.

### 3.2 Intraoperative imaging, dose calculation and in situ dosimetry

**FIGURE 7:**
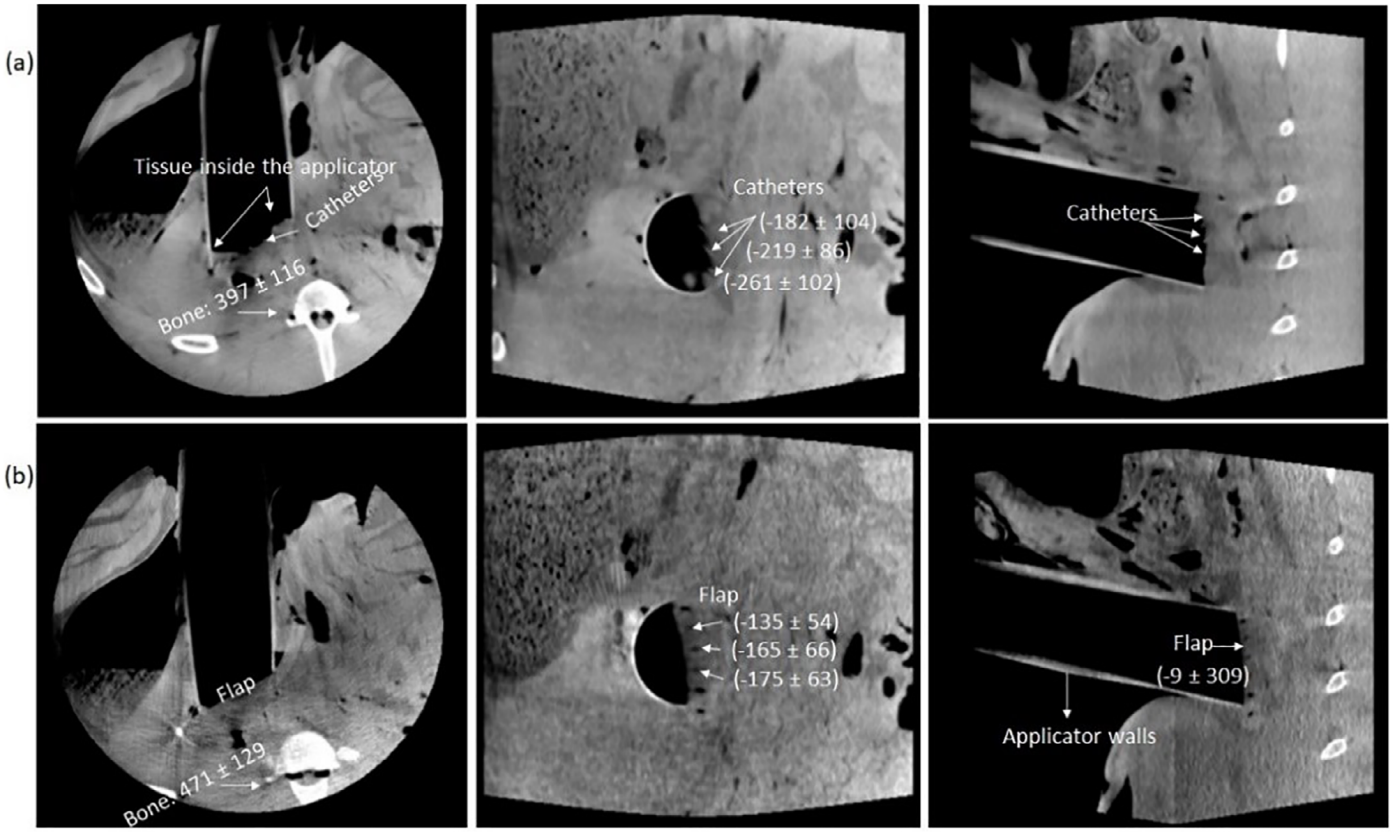
Intraoperative ImR CBCT images of the porcine cadaver with two TLD‐based dosimetry systems and the IOERT applicator in place. From left to right, the images correspond to the transversal, coronal, and sagittal planes. The CBCT numbers of the flap and catheters are shown in parentheses together with their standard deviations (*k* = 1), as extracted in Eclipse™. (a) Set‐up with three catheters loaded with TLDs and wrapped in a sterile surgical foil, (b) set‐up with the flap material filled with three catheters loaded with TLDs.

**FIGURE 8:**
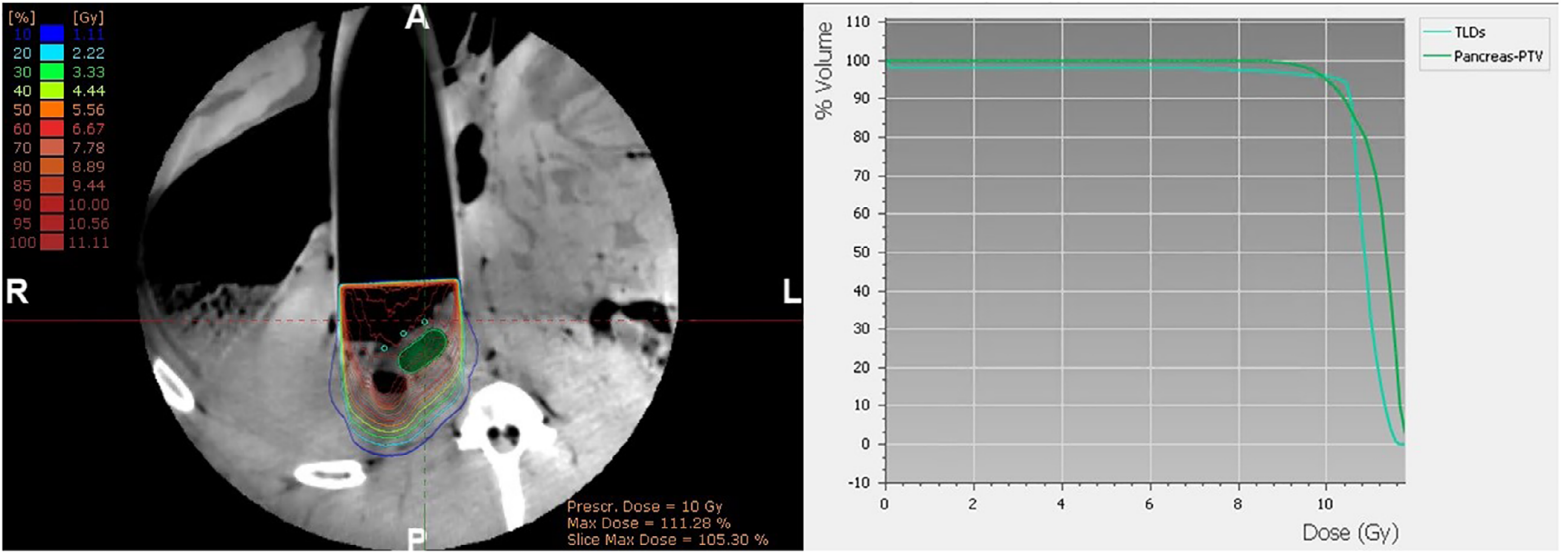
Dose distribution in the transversal plane for the catheter set‐up configuration, including the resulted DVH for the PTV and the TLD‐catheter segmentations.

**FIGURE 9:**
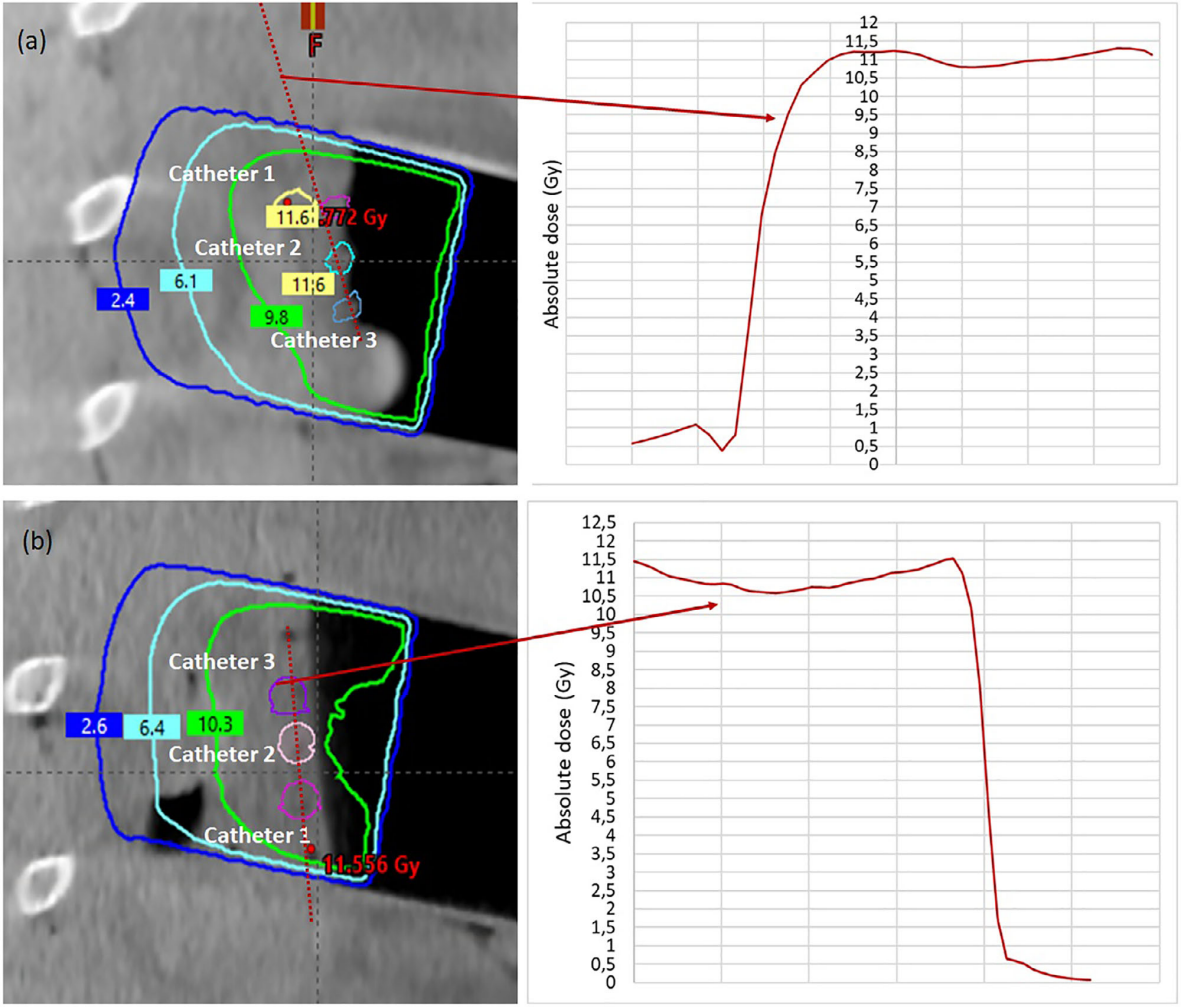
Dose distributions in sagittal plane. The contours of the catheters are also presented together with the extracted dose profiles pointed out with the red arrow. The dose calculation starts within the applicator due to tissue inside it. (a) Catheter set‐up and (b) flap set‐up.

Figure 7 presents the intraoperative CBCT images, with CBCT numbers (shown in parentheses) for the catheters, the flap, catheters within the flap and bone. The applicator walls were clearly visible, allowing accurate placement of the virtual applicator in Radiance. Both the catheters and the flap were distinguishable. Notably, the holes in the flap without TLDs appeared black, providing high contrast with the surrounding material. On the contrary, the filled holes with TLDs appeared less distinct with reduced contrast. However, soft tissue contrast was insufficient to clearly identify the pancreas PTV or the major vessels in its vicinity, such as the superior mesenteric artery, celiac trunk and aorta, which are of primary clinical interest in postpancreatic resection applications. Therefore, a pancreatic PTV was contoured based on its known location beneath the dosimetry set‐up. The mean CBCT number values for the pancreatic PTV contours were −24 ± 55 HU for the flap set‐up (Pr‐120 kV_p_) and −70 ± 60 HU for the catheter set‐up (Pr‐110 kV_p_), whereas the pancreatic tissue contour in the preoperative CT scan resulted in 35 ± 60 HU. For the same bone ROI depicted in Figure 7, the CT scan yielded 469 ± 156 HU, while the intraoperative ImR CBCT scans produced 397 ± 116 HU (Pr‐110) and 471 ± 129 HU (Pr‐120) (Figure 7a, b). Figure 8 shows the dose distributions and the corresponding dose–volume histogram (DVH) for the catheter set‐up. Table 2 summarizes the TLD measurement results alongside the corresponding mean contour doses calculated inside the catheters for both dosimetry set‐ups, including the percentage deviation between measured and calculated values. Radiance‐calculated MU agreed with manually evaluated values, differing by only 1 MU (787 MU). Figure 9 illustrates the extracted dose profiles along the TLD positions. According to these profiles, the dose values ranged between 10.5 Gy and 11.5 Gy in the vicinity of the TLDs, consistent with the measured values. The contours for each catheter were drawn with a diameter of 4 mm, larger than the physical catheter diameter of 1 mm, to ensure adequate sampling coverage. For individual catheters, the calculated dose deviated by approximately 3% from the measured dose, well within the clinically accepted tolerance of ±5% for absorbed dose determination and in vivo dose verification.^33^, ^34^, ^35^ The mean dose obtained by averaging the doses across all three catheters showed excellent agreement with the corresponding measured mean dose for both set‐ups, with deviations below 1%.

**TABLE 2:** Measured and calculated TLD doses inside the catheters for the two dosimetry set‐ups.

Measured TLD dose (Gy ± SD (*k* = 1))			Calculated mean TLD‐contour dose (Gy ± SD (*k* = 1))			
	Flap set‐up	Catheter set‐up	Flap set‐up	% dev	Catheter set‐up	% dev
**Catheter 1**	11.47 ± 0.37	11.08 ± 1.48	11.15 ± 0.45	−2.79	10.86 ± 0.27	−1.99
**Catheter 2**	11.28 ± 0.29	10.43 ± 0.38	10.96 ± 0.32	−2.83	10.76 ± 0.86	3.16
**Catheter 3**	10.75 ± 0.29	11.19 ± 0.40	11.03 ± 0.25	2.60	11.17 ± 0.25	−0.18
**Mean dose**	11.13 ± 0.55	10.90 ± 0.97	11.05 ± 0.20	−0.72	10.93 ± 0.31	0.28

*Note*: The mean dose refers to the average dose across all three catheters. The dose for each catheter corresponds to the mean value of the five TLD measurements within that catheter.

## 4 DISCUSSION

In this work, we evaluated the developed IOERT workflow using a porcine cadaver model. Imaging was performed with the ImR system (medPhoton), MC dose calculations were carried out in Radiance TPS version 4.08.2 and in situ dose measurements were obtained with TLD‐100 detectors.^34^ The experimental validation was successfully completed and provided valuable insight into the feasibility of clinical translation using the available equipment. Regarding the calibration and ImR imaging, accurate calibration tables with acceptable CBCT number uniformity are essential. Otherwise, the LUT generated in Radiance for MC dose calculations may differ for the same imaging protocol when different acquisition modes are used. In the framework of IOERT imaging, SFOV scans are preferable, as they reduce scatter contributions from surrounding surgical tools. Moreover, full abdominal coverage is not required for IOERT treatment planning. The resulting CBCT numbers showed good agreement with the corresponding reference data.

The limitations of this study were primarily related to external factors associated with the experimental set‐up. In general, the pancreas is difficult to identify on diagnostic CT images and contrast agents were not used in this work. In addition, not all clinical surgical accessories could be employed, such as anesthesia‐related equipment (e.g., ventilator or infusion stands), because the experiment was performed in the brachytherapy suite rather than in the fully equipped OR. Consequently, the pancreas was not resected to create a tumor bed. Instead, the pancreatic tissue remained in situ and the TLDs were positioned on its surface. However, this enabled a direct comparison of pancreatic HU values between the reference preoperative CT and the intraoperative CBCT images. Lower HU values were observed for the pancreatic PTV in the intraoperative CBCT scans, likely due to differences in photon attenuation under open surgical geometry compared with the closed‐surface geometry of the preoperative CT. These findings highlight the significant impact of geometric conditions on the resulting CBCT numbers.

Limited soft‐tissue contrast was obtained; however, this did not compromise visualization of the applicator walls or the TLD set‐ups. Accurate applicator placement, TLD contouring, and dose delivery within clinically accepted tolerances (±5%) were achieved. To improve soft‐tissue contrast, the administration of contrast agents represents a potential option for future intraoperative irradiations. Additional strategies may include dual‐energy imaging, synthetic CT generation, and deep learning–based approaches for image quality enhancement.^36^, ^37^, ^38^ However, these are not currently fully supported by the equipment used in this study.

Furthermore, dose calculation was influenced by the configuration of the virtual applicator in Radiance. Because tissue extension into the applicator was observed, dose calculations needed to be performed within the applicator. The starting position of dose calculation engine in Radiance relative to the tissue interface plays a significant role in the resulting dose distributions affecting the dose scored in the contours. In Radiance, the tool that enables dose calculation within the applicator vertically shifts the starting point by 4 cm. However, such a shift was not required at the present study. Instead, the virtual applicator was repositioned downwards to align the starting point of the dose calculation closer to the tissue interface. This adjustment slightly compromises the dose distribution at the applicator edges by reducing the lateral spread of the isodose curves. Nevertheless, within the scope of this study, this effect was considered negligible. in situ dosimetry demonstrated discrepancies of up to 3% from the calculated mean contour doses of each individual catheter, and less than 1% from the overall surface dose. The dose profiles extracted along the catheter axes also agreed with the range of dose values measured by the TLDs.

In general, several factors contribute to the overall experimental uncertainty. Firstly, the size of the delineated contours was larger than the physical one due to sampling coverage constraints. Furthermore, the CBCT‐related effects such as ImR calibration and CBCT number accuracy and variability within the calculation volume. In addition, the reliable visualization of the TLD positions, and consequently, their accurate contouring in the TPS, depends on image resolution and quality. This, in turn, affects the spatial alignment between calculated and measured dose points. The dose difference resulting from a positional uncertainty of a few mm in the contoured TLD depends on the local dose gradients. MC related uncertainties further contribute through statistical uncertainty governed by the user‐defined percentile tolerance, as well as uncertainties propagated from the generated beam model. In this study, a 1% tolerance was applied; tolerances of up to 5% can be selected in Radiance. The beam model was commissioned with 3% dose difference and 3 mm distance‐to‐agreement gamma criteria.^32^ Measurement‐related uncertainties arise from TLD calibration, incorporating both type A and type B components associated with correction factors, signal readout, and reproducibility. The overall uncertainty in our previous work was estimated to be approximately 3% for the 10 Gy dose level.^31^ Overall, the dominant contributors to the propagated uncertainty are the TLD calibration uncertainties,^31^, ^39^ volume‐contouring effects, CBCT imaging effects (calibration, CBCT number reliability, and image quality), and the MC related factors (percentile tolerance and beam model).

It is also important to acknowledge that differences between the experimental porcine model and clinical patients may introduce additional challenges. In clinical scenarios, variations in patient Body Mass Index (BMI), free breathing, and surgical conditions can affect CBCT image quality. Moreover, the presence of additional surgical tools and equipment in the operating room may further complicate the imaging set‐up by increasing scatter and reducing image clarity. Our pilot phase clinical trial of twenty patients will therefore be crucial to optimize protocols, gain practical experience, and finalize workflow procedures. Clinical implementation may also benefit from the adoption of x‐ray compatible surgical tools to optimize intraoperative imaging quality.

**FIGURE 10:**
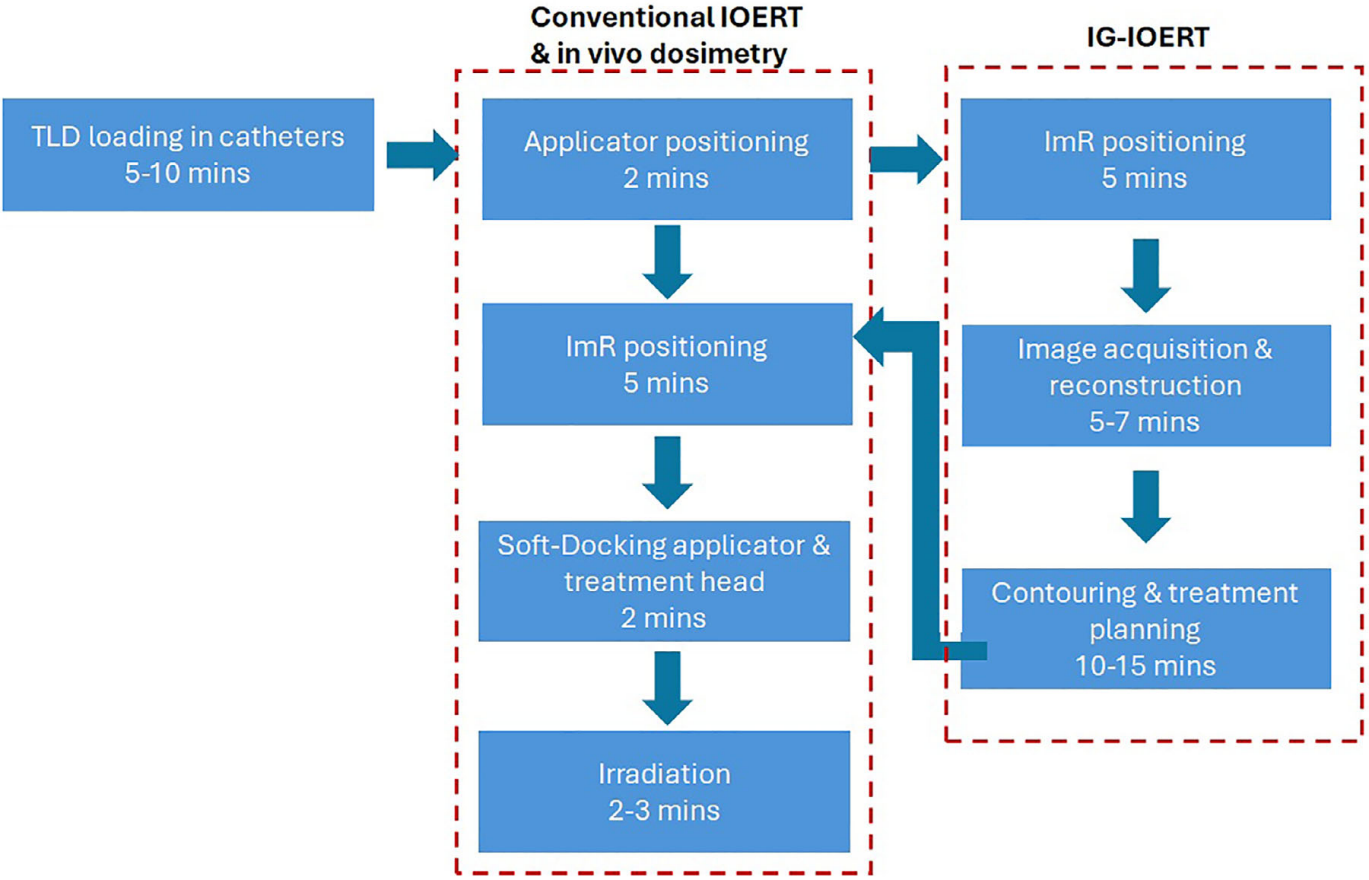
A schematic representation of the workflow maximum required times.

Finally, the total additional time required for the pancreatic IOERT workflow in a real‐time clinical scenario is estimated to be 50 min for imaging and treatment planning, compared to 20–25 min for the conventional pancreatic IOERT workflow with in vivo dosimetry. The longer time is mainly associated with CBCT acquisition, contouring and MC dose calculation, which may require 1–10 min depending on image artifacts, applicator diameter, and the selected calculation starting point. In some cases, repeated CBCT scans are necessary when applicator positioning requires adjustment. These time requirements are comparable to those reported for low‐kV X‐ray breast IORT or flap‐based ^192^Ir IORT workflows at our institution. A schematic representation of the time required for each workflow (with and without image guidance (IG‐IOERT) is provided in Figure 10.

Successful clinical translation requires coordinated interdisciplinary communication and comprehensive training of all involved staff, including surgeons, nurses, anesthesiologists, and radiation oncologists. Respiratory motion management is also critical, as careful anesthetic control is necessary to minimize motion artifacts that could degrade CBCT image quality. In summary, the present study successfully validated the developing pancreatic IOERT workflow in a porcine cadaver model, demonstrating that imaging, dose calculation, and in vivo dosimetry can be effectively integrated with the current equipment. Additionally, the observed agreement between TLD measurements and calculated doses, within clinically acceptable tolerances provides a solid basis for future clinical translation of image‐based IOERT for pancreatic cancer.

## 5 CONCLUSION

We successfully simulated the complete developed pancreatic IOERT clinical workflow (image‐based dose calculations and in vivo dosimetry) on a porcine cadaver. ImR imaging provided reliable CBCT numbers and sufficient image quality for accurate applicator positioning and treatment planning. Dose calculations agreed with in situ measurements within 3%, supporting further clinical implementation and patient‐specific optimization in pancreatic IOERT.

## AUTHOR CONTRIBUTIONS

Charoula Iliaskou: Wrote the manuscript, study design, organization and participation of the experiments and data analysis. Approves the final version of the manuscript. Mark Gainey, Michael Kollefrath, Andreas R. Thomsen, Dietrich A. Ruess and Dimos Baltas: study design, participation in the experiment, revision of the manuscript and approval of the final version. Siegmar Kuhn: participation in the contouring of the region of interest on the CT image and approval of the final version of the manuscript. Anca‐Ligia Grosu approves the final version of the manuscript.

## CONFLICT OF INTEREST STATEMENT

The authors declare no conflicts of interest.
